# Notes on *Carex* (Cyperaceae) from China (X): new species and variety of sections *Clandestinae* and *Rhomboidales*

**DOI:** 10.3897/phytokeys.281.211013

**Published:** 2026-11-04

**Authors:** Yi-Fei Lu, Wei-Qiang Qin, De-Chang Meng, Xin-Jie Zhao, Hong Wang, Xiao-Feng Jin

**Affiliations:** 1 College of Forestry (College of Life Sciences), Zhejiang A&F University, Hangzhou 311300, Zhejiang, China College of Forestry (College of Life Sciences), Zhejiang A&F University Hangzhou China; 2 School of Science and Engineering, Zhangjiajie College, Zhangjiajie 427000, Hunan, China School of Science and Engineering, Zhangjiajie College Zhangjiajie China; 3 College of Horticulture and Landscape Architecture, Zhongkai University of Agriculture and Engineering, Guangzhou 510225, Guangdong, China College of Horticulture and Landscape Architecture, Zhongkai University of Agriculture and Engineering Guangzhou China; 4 Herbarium BAZI, Bazi Collection & Botanical Garden, Mengzi 661199, Yunnan, China Herbarium BAZI, Bazi Collection & Botanical Garden Mengzi China; 5 China United Engineering Co., Ltd., Hangzhou 310052, Zhejiang, China China United Engineering Co., Ltd. Hangzhou China

**Keywords:** *
Carex
*, endemic species, morphological traits, nomenclature and taxonomy

## Abstract

*Carex* L. is one of the largest angiosperm genera and the most species-rich genus of flowering plants in China. Three new species and one new variety belonging to *Carex* sect. *Clandestinae* and sect. *Rhomboidales* are described and illustrated. *Carex zhangjiajieensis* (sect. *Clandestinae*) differs from the morphologically similar species *C. shuchengensis* in having lateral peduncles exserted from bract sheaths, utricles slightly longer than or subequal to pistillate glumes and ventrally veinless, and nutlet beaks that are erect and ca. 0.2 mm long. Within sect. *Rhomboidales*, *C. glaucispicata* (**sp. nov**.) differs from *C. cheniana* by its dwarf habit, shorter pistillate spikes, and narrower leaves. *Carex liboensis* (**sp. nov**.) differs from the related species *C. austrosinensis* in having rather densely flowered lateral spikes, lateral peduncles enclosed in bract sheaths, and nutlets shallowly constricted at the three middle angles. Carex canina var. pubiutriculata (**var. nov**.) differs from the autonymic variety in having both pubescent utricles and bract sheaths.

## Introduction

The genus *Carex* comprises approximately 2,300 species and is the largest genus in Cyperaceae (Roalson et al. 2021; Lu et al. 2025; Jiménez-Mejías et al. 2026). It is exceptionally species-rich, broadly distributed, and occupies a wide range of habitats (Dai et al. 2010; Jin and Zhang 2020; Roalson et al. 2021). Morphologically, *Carex* was traditionally divided into four groups based on inflorescence and spike architecture: (1) the species with a single, exclusively androgynous spike; (2) those with numerous androgynous spikes arranged in a paniculate inflorescence, where the basal spike arises from a utricle-like cladoprophyll; (3) those with numerous sessile, bisexual spikes densely aggregated into a spicate inflorescence, with cladoprophylls usually reduced or absent and nutlets bearing two stigmas; and (4) those with few spikes arranged in a racemose inflorescence, bearing sheathing cladoprophylls (Chang and Yang 1976; Chater 1980; Egorova 1999). Kükenthal (1909) relied heavily on these characters to subdivide the genus into four subgenera, although subg. *Primocarex* was rejected by several subsequent caricologists (Kreczetowicz 1935; Mackenzie 1935; Ohwi 1936; Dai et al. 2000, 2010).

With the advent of molecular phylogenetics, it became evident that most traditionally recognized subgenera and sections within *Carex* were non-monophyletic and failed to represent natural evolutionary lineages (Jiménez-Mejías et al. 2016; Martín-Bravo et al. 2019; Roalson et al. 2021). Consequently, the systematics of the genus is currently undergoing profound revision. Recently, the Global *Carex* Group proposed an updated framework integrating morphological and molecular evidence, dividing *Carex* into six subgenera, further subdivided into 62 formally named sections and 49 informal groups (Villaverde et al. 2020; Roalson et al. 2021).

China harbors an exceptionally rich diversity of *Carex*, hosting more than 600 species, including those formerly placed in *Kobresia* (now subsumed within *Carex*) (Li and Zhang 2024). “Flora Reipublicae Popularis Sinicae” and “Flora of China” provided comprehensive botanical syntheses of earlier taxonomic treatments (Dai et al. 2000, 2010). Intensive field exploration over recent decades has led to the discovery and publication of numerous new taxa. Ongoing taxonomic research and specimen revisions have resulted in the description of 27 new *Carex* species (Jin et al. 2011; Zhao et al. 2011; Jin et al. 2012, 2015a, 2015b; Zhou and Jin 2014; Chen and Jin 2015; Lu and Jin 2017, 2018, 2022; Lu et al. 2020, 2024; Lu et al. 2022, 2023, 2025; Qiu et al. 2024), as well as dedicated taxonomic monographs for sections *Rhomboidales* and *Mitratae* (Jin and Zheng 2013; Jin and Lu 2024). Herein, three additional new species and one new variety are described in sections *Clandestinae* and *Rhomboidales*, respectively.

## Materials and methods

Specimens of the three new species and one variety were collected from four different regions in China. Careful examination of utricle and nutlet morphology placed these taxa in *Carex* sections *Clandestinae* and *Rhomboidales*, respectively. Specimens collected from Mt. Tianmenshan (western Hunan Province) belong to sect. *Clandestinae*, characterized by spathe-like bracts and densely hispidulous utricles. Material collected from Sichuan, Guizhou, and Zhejiang provinces exhibits rhombic-ovoid nutlets with constricted middle angles, placing them in sect. *Rhomboidales*.

The herbarium specimens deposited in ACM, FNU, BM, E, HAST, HHBG, HTC, HZU, IBK, IBSC, IFP, K, KUN, KYO, LE, N, OKAY, P, PE, SAP, SDNU, SZ, TI, WUK, ZJFC, and ZM were examined. All type specimens in these herbaria were carefully checked, compared, and photographed. Other related species of *Carex* sect. *Clandestinae* and sect. *Rhomboidales* were examined online images from the Kew Herbarium Catalogue (http://apps.kew.org/herbcat/gotoHomePage.do) and JSTOR Global Plants (https://plants.jstor.org/).

Morphological descriptions were compiled directly from mature, fully developed specimens. Measurements of leaf width, spike dimensions, bract sheath length, glume lengths (staminate and pistillate), and utricle and nutlet dimensions were performed on reproductive individuals. For infraspecific taxonomy, two ranks are recognized: subspecies and varieties. The subspecies rank is applied to taxa that show distinct geographic or altitudinal partitioning, whereas the variety rank is applied to distinct morphological variants without geographic isolation.

## Taxonomic treatment

### 1. Carex zhangjiajieensis

Taxon classificationPlantaePoalesCyperaceae

Y.F.Lu, W.Q.Qin & X.F.Jin sp. nov.

305D6E54-BAC9-5753-8647-95E0146B7CCB

urn:lsid:ipni.org:names:77484426-1

Figs 1A–E, 2A–F

#### Diagnosis.

This new species is similar to *Carex shuchengensis* S.W.Su in having short rhizomes, leathery leaves, and thick spikes, but differs in having peduncles of pistillate spikes exserted from bract sheaths, utricles slightly longer than pistillate glumes or nearly equal in length, ventrally veinless, nutlets with a ca. 0.2 mm long erect beak at the apex.

**Figure 1.**
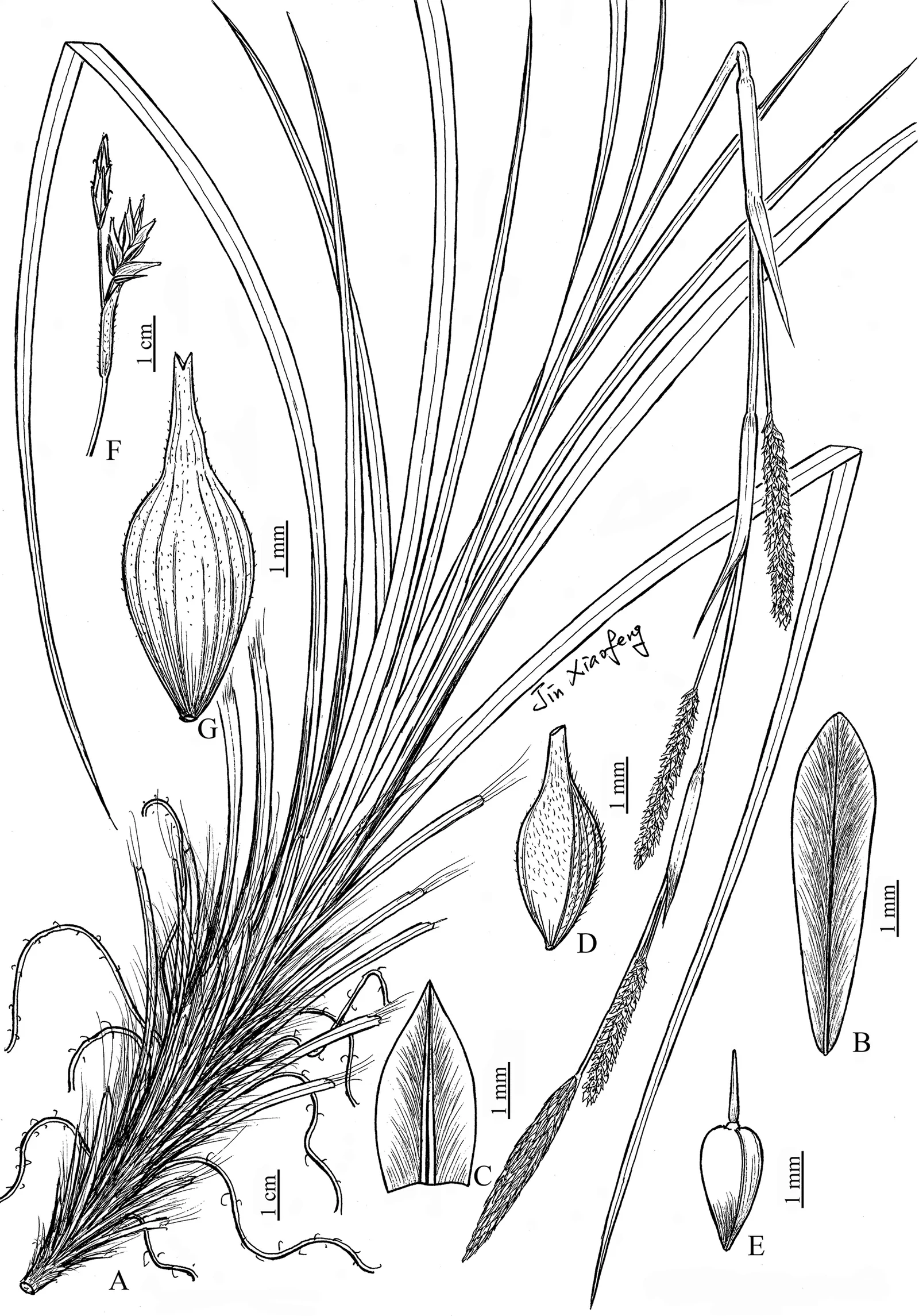
*Carex tianmenshanica* sp. nov. (**A–E**) and C. canina var. pubiutriculata var. nov. (**F, G**). **A**. Habit; **B**. Staminate glume; **C**. Pistillate glume; **D**. Utricle; **E**. Nutlet; **F**. Upper part of inflorescence; **G**. Utricle (drawn by Xiao-Feng Jin from the holotype).

**Figure 2.**
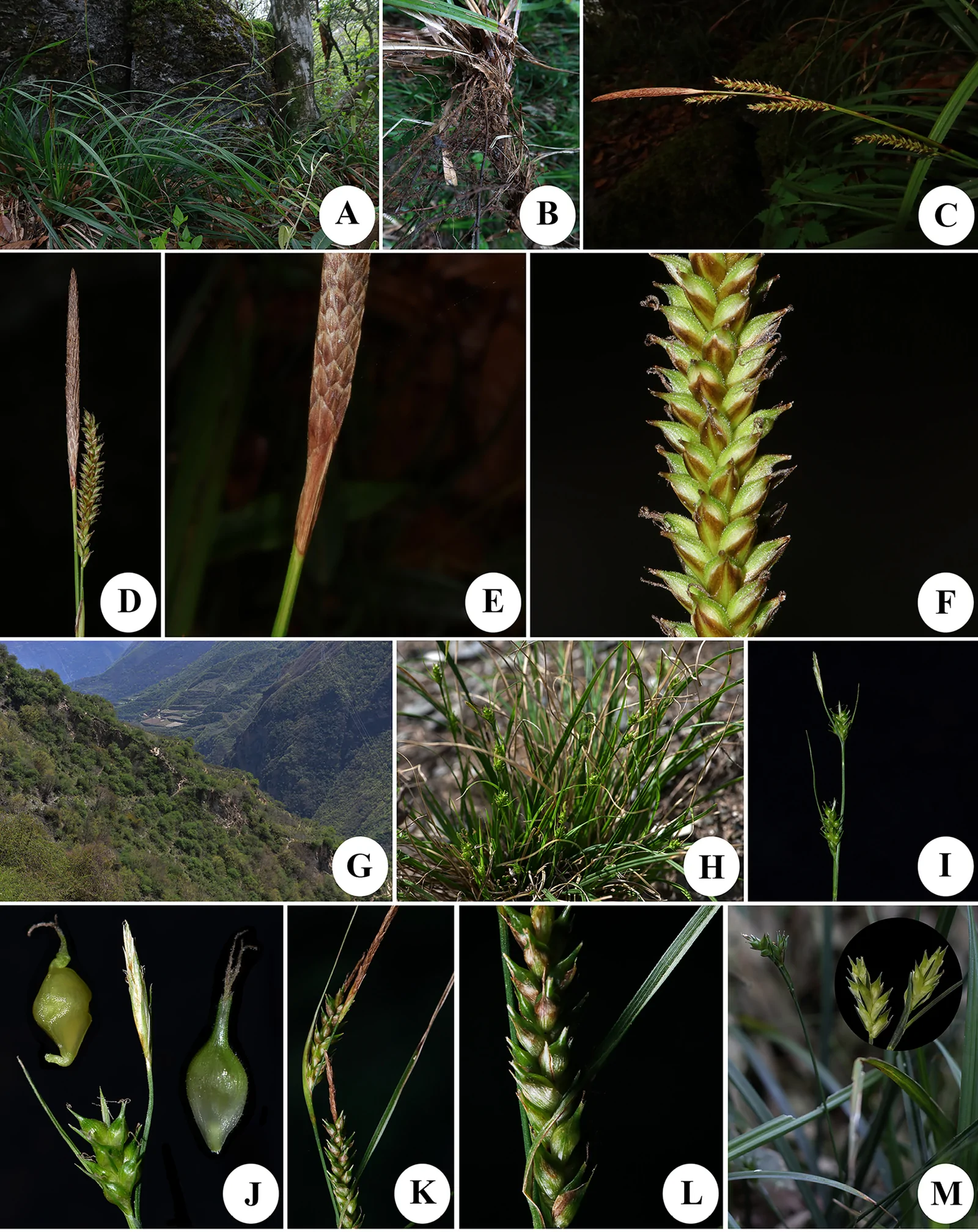
Photos of *Carex tianmenshanica* (**A–F**), *C. glaucispicata* (**G–J**), *C. liboensis* (**K, L**) and C. canina var. pubiutriculata (**M**). **A**. Habitat; **B**. Lower part of plant, showing rhizome; **C**. Inflorescence; **D**. Terminal spike and uppermost spike; **E**. Staminate spike, showing staminate glumes; **F**. Pistillate spike, showing pistillate glumes and utricles; **G**. Habitat; **H**. Habit; **I**. Inflorescence; **J**. Terminal spike and uppermost spike, together with utricle and nutlet; **K**. Terminal spike and two uppermost spikes; **L**. Lateral spike, showing pistillate glumes and utricles; **M**. Habit, together with spikes.

#### Type.

China • Hunan: Zhangjiajie City, Yongding Dist., Mt. Tianmenshan, Yuhufeng; alt. 1,450 m; in roadside grass under forest; 27 April 2024; *X. F. Jin, Y. F. Lu & W. Q. Qin 5372* (holotype: ZJFC; isotypes: IBK, ZM).

#### Description.

Herbs perennial. Rhizomes woody, stiff, obliquely ascending. Culms central, loosely caespitose, 25–45 cm tall, obtusely trigonous, base with dark brown fibrous sheath. Leaves longer to shorter than culms; blades flat, leathery, 3.5–8 mm wide, apex long-acuminate, scabrous on upper lower surface and margins. Bracts spathe-like, shorter than inflorescence, apex awned, base sheathing; sheaths 1.2–3 (–4) cm long. Spikes four or five, racemose, remote; terminal spike staminate, clavate-cylindrical, 3–5 cm long, 3–4.5 mm wide, densely flowered, base pedunculate; lateral spikes pistillate, cylindrical, 2–5 cm long, 3–5.5 mm wide, densely flowered, base with peduncles exserted from sheaths. Staminate glumes oblanceolate, pale brown, upper part brown, pale yellow laterally, 8–9 mm long, apex obtuse, with 1-veined brown costa dorsally. Pistillate glumes broadly ovate or ovate, upper part pale brown, lower part pale yellow, 4–4.5 mm long, apex acute and mucronate, with 3-veined green and scabrid costa dorsally. Utricles pale yellow, obovoid (beak excluded), obtusely trigonous, 4–4.5 mm long, slightly longer than pistillate glumes, or nearly equal in length, dorsally distinctly thinly veined, ventrally veinless, densely hispidulous, base attenuate, apex abruptly contracted into a ca. 1.2 mm long beak, orifice obliquely truncate. Nutlets tightly enveloped, pale yellow, obovoid, trigonous, 2–2.2 mm long, with three sides slightly concave below, apex abruptly contracted into a ca. 0.2 mm beak; style erect, base slightly thickened; stigmas three.

#### Etymology.

The specific epithet “*zhangjiajieensis*” refers to the type locality of this new species.

#### Phenology.

Flowering and fruiting occur from late April to early May.

#### Additional specimens examined.

China • Hunan: Zhangjiajie City, Yongding Dist., Mt. Tianmenshan, 29.05163°N, 110.48356°E, alt. 1,402 m, on roadside slope under forest, 8 May 2026, *Y. F. Lu, Q. Q. Qin, L. L. Wang & W. Q. Qin 1393* (IBK, ZJFC, ZM), • 29.05469°N, 110.47978°E, alt. 1,021 m, on roadside slope under forest, 8 May 2026, *Y. F. Lu, Q. Q. Qin, L. L. Wang & W. Q. Qin 1400* (ZJFC, ZM); • Wulingyuan, Mt. Tianzishan, 29.37355°N, 110.46300°E, alt. 910 m, on limestones, 7 May 2026, *Y. F. Lu, Q. Q. Qin, L. L. Wang & W. Q. Qin 1387* (ZJFC, ZM).

#### Conservation status.

The new species is currently known from a few collections from Mts. Tianmenshan and Tianzishan in Zhangjiajie City, Hunan Province. All known localities are situated within scenic areas subject to substantial human activity, and tourism-related disturbance and roadside vegetation management may pose potential threats to the species. However, the extent to which these activities affect its habitat and populations remains unclear. Further field surveys are needed to determine population size and trends, assess habitat condition, and establish whether continuing declines are occurring. The species is therefore provisionally assessed as Data Deficient (DD) under the IUCN Red List Categories and Criteria (IUCN 2024).

#### Notes.

The new species is readily distinguished within *Carex* sect. *Clandestinae* by its robust habit, rhizomes, and spikes. Morphologically, it is similar to *Carex shuchengensis* S.W.Su, known from western Anhui Province, but differs in having peduncles of lateral pistillate spikes exserted from bract sheaths, utricles slightly longer than or subequal to pistillate glumes and adaxially veinless, and nutlets with an erect beak ca. 0.2 mm long at the apex. Additionally, *Carex dayongensis* Z.P.Wang, an endemic species of sect. *Clandestinae* from western Hunan Province, is distinguished from the new species in having leaf blades 1–2 mm wide and utricles with conspicuous distinctive veins and an extremely short beak less than 0.5 mm long.

### 2. Carex glaucispicata

Taxon classificationPlantaePoalesCyperaceae

D.C.Meng & X.F.Jin sp. nov.

E5F3E1F2-A006-5600-91B9-8C119BC0D285

urn:lsid:ipni.org:names:77484427-1

Figs 2G–J, 3A–E

#### Diagnosis.

This new species is similar to *Carex cheniana* Tang & F.T.Wang ex S.Yun Liang in having central culms and glaucous leaves and spikes, but can be distinguished by shorter pistillate spikes, 1–2 cm long, leaves 1.5–2 mm wide, plant dwarf.

**Figure 3.**
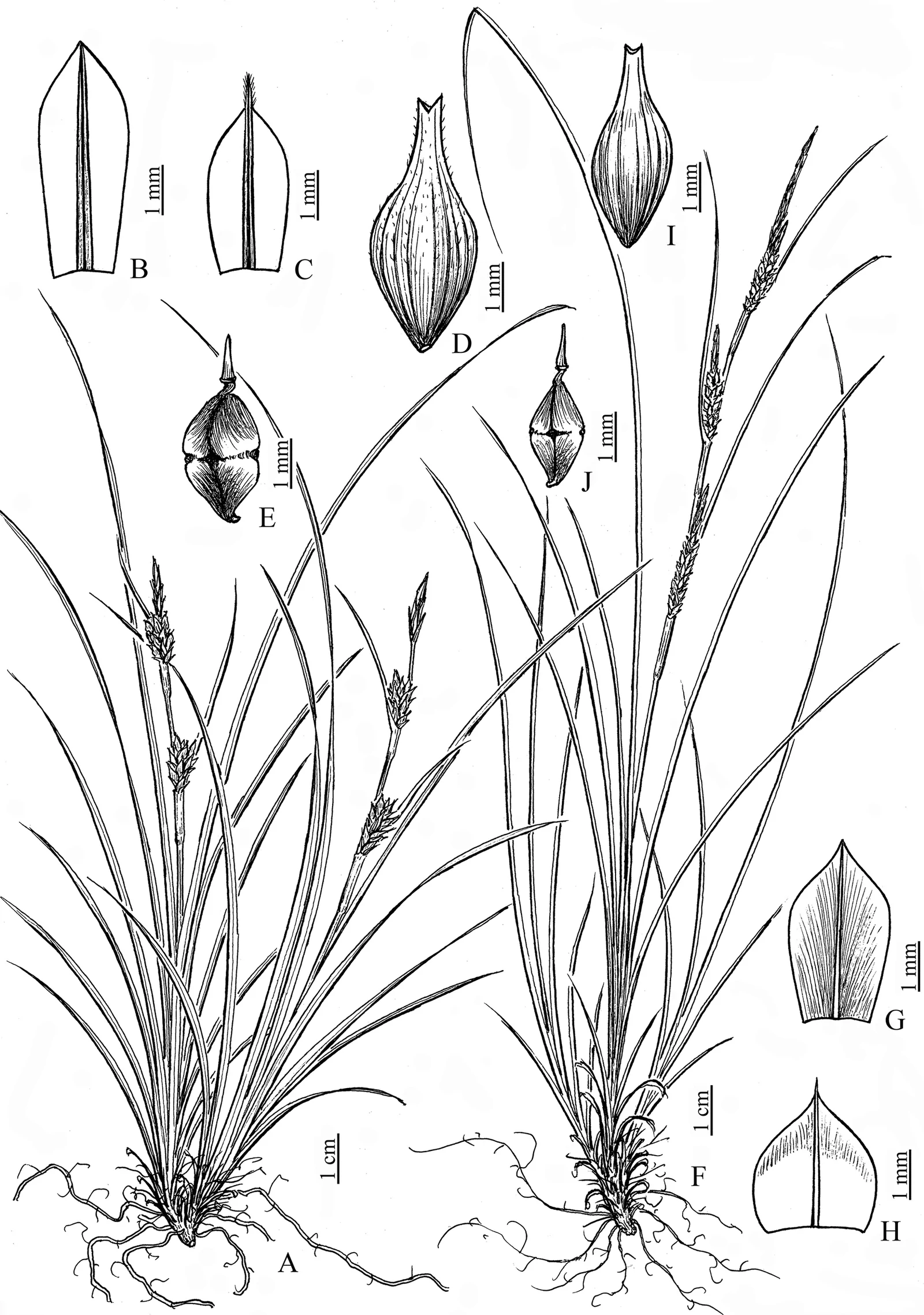
*Carex glaucispicata* sp. nov. (**A–E**) and *C. liboensis* sp. nov. (**F–J**). **A, F**. Habit; **B, G**. Staminate glume; **C, H**. Pistillate glume; **D, I**. Utricle; **E, J**. Nutlet (drawn by Xiao-Feng Jin from the holotype).

#### Type.

China • Sichuan: Jiuzhaigou Xian, Nanping Town, near Gangyan Tunnel; 33.282460, 104.220800; alt. 1,580 m; in roadside grass; 18 April 2023; *D. C. Meng 189408* (holotype: ZJFC; isotype: ZM).

#### Description.

Herbs perennial. Rhizomes short, woody, stiff. Culms central, loosely caespitose, 16–30 cm tall, obtusely trigonous, slightly scabrous, base with dark brown fibrous sheath. Leaves longer than culms; blades flat, nearly leathery, (1–)1.5–2 mm wide, apex long-acuminate, scabrous on lower surface and margins. Bracts shortly leaf-like, lowers longer than spikes, uppers nearly equal to spikes, base sheathing; sheaths 4–12 mm long. Spikes three or four, racemose, remote; terminal spike staminate, short-clavate, 1–2 cm long, 2.5–3 mm wide, densely flowered, base pedunculate; lateral spikes pistillate, short-cylindrical, 1–2 cm long, 4.5–6 mm wide, densely flowered, sometimes apex with a few staminate flowers, base with peduncles enclosed in sheaths. Staminate glumes obovate-lanceolate, glaucous or white-green, 4.5–5 mm long, apex acute, with 3-veined green costa dorsally. Pistillate glumes obovate, glaucous or white-green, 4–4.5 mm long, apex acuminate, with 3-veined green costa dorsally excurrent into a 0.7–1 mm long scabrous awn. Utricles pale yellow, obovoid (beak excluded), obtusely trigonous, 5–5.5 mm long, longer than pistillate glumes, distinctly thinly veined, pubescent, base attenuate, apex gradually contracted into a ca. 1.5 mm long beak, margin pubescent, orifice 2-lobed with short teeth. Nutlets tightly enveloped, pale yellow, rhombic-ovoid, trigonous, 2.5–2.8 mm long, with three angles constricted at middle, sides concave above and below, base with a short and curved stipe, apex abruptly contracted into a short, coiled beak; style base thickened; stigmas three.

#### Etymology.

The specific epithet “*glaucispicata*” refers to the staminate and pistillate spikes of this new species, which are pale green or glaucous when dried.

#### Phenology.

Flowering and fruiting occur in mid-April.

#### Conservation status.

*Carex glaucispicata* is currently known only from the type locality. In the absence of comprehensive field surveys, its geographic range, population size and trends, and potential threats remain poorly known. The available information is therefore insufficient to determine whether the species meets any of the IUCN criteria for a threatened category. The species is provisionally assessed as Data Deficient (DD) following the IUCN Red List Categories and Criteria (IUCN 2024).

#### Notes.

Based on dried herbarium specimens, the leaves and spikes of the new species are pale green, rendering it superficially similar to *Carex cheniana* Tang & F.T.Wang ex S.Yun Liang. However, these two species differ markedly in plant size, leaf width, and spike length. A detailed morphological comparison between them is presented in Table 1.

**Table 1.** Morphological comparison between *Carex glaucispicata* and *C. cheniana*.

**Character**	***C. glaucispicata***	***C. cheniana***
Culms	16–30 cm tall, obtusely trigonous	30–65 cm tall, trigonous
Leaf blades	1.5–2 mm wide	4–11 mm wide
Bracts	Shortly leaf-like, with sheath 4–12 mm long	Leaf-like, with sheath 15–30 mm long
Staminate spikes	1–2 cm long, 2.5–3 mm wide	1–3.5 cm long, ca. 3 mm wide
Pistillate spikes	Short-cylindrical, 1–2 cm long, 4.5–6 mm wide	Cylindrical, 2.5–7 cm long, 6–8 mm wide
Staminate glumes	4.5–5 mm long, apex acute	10–12 mm long, apex scabrous, long-awned
Pistillate glumes	4–4.5 mm long, apex with a scabrous awn 0.7–1 mm long	8–9 mm long, apex with a scabrous awn ca. 3.5 mm long
Utricles	5–5.5 mm long, longer than pistillate glumes	6.5–8 mm long, shorter than pistillate glumes
Nutlets	2.5–2.8 mm long	3–3.5 mm long

### 3. Carex liboensis

Taxon classificationPlantaePoalesCyperaceae

X.J.Zhao & X.F.Jin sp. nov.

C5265733-61D9-5DAA-9E67-AE315BB8940B

urn:lsid:ipni.org:names:77484428-1

Figs 2K, 2L, 3F–J

#### Diagnosis.

This new species is similar to *Carex austrosinensis* Tang & F.T.Wang ex S.Yun Liang in having central culms, leaf-like bracts and brown-green utricles but differs by lateral spikes rather densely flowered, base with peduncles enclosed in bract sheaths, nutlets with three angles shallowly constricted at middle.

#### Type.

China • Guizhou: Libo Xian, Shishang Forest Scenic Area; 25.273400, 107.895980; on limestone under forest; 22 March 2024; *X. J. Zhao 2024032201* (holotype: ZJFC; isotype: ZM).

#### Description.

Herbs perennial. Rhizomes short, woody, stiff. Culms central, densely caespitose, 20–40 cm tall, trigonous, scabrous, base with brown or yellow-brown fibrous sheath. Leaves longer than culms, sometimes nearly equal in length; blades flat, nearly leathery, 1–1.5 mm wide, scabrous on both surfaces and margins. Bracts leaf-like, longer than spikes, lowest far longer than others, base sheathing; sheaths 4–20 mm long. Spikes four, racemose, remote; terminal spike staminate, thinly cylindrical, 2–3 cm long, 1–1.5 mm wide, densely flowered, base with a slender peduncle; lateral spikes androgynous, cylindrical, 2–3.2 cm long, 3.5–4 mm wide, staminate part 0.5–2 cm long, pistillate part 1–2 cm long, base with peduncles enclosed in sheaths. Staminate glumes obovate, brown, laterally pale brown, 3.5–4 mm long, apex acute, with 1-veined dark brown costa dorsally. Pistillate glumes broadly ovate, pale brown, ca. 3 mm long, apex mucronate, with 1-veined dark brown costa dorsally. Utricles brown-green, obovoid (beak excluded), obtusely trigonous, 5–5.5 mm long, longer than pistillate glumes, distinctly thinly veined, glabrous, base attenuate, apex gradually contracted into a ca. 1 mm long beak, orifice 2-lobed with short teeth. Nutlets tightly enveloped, castaneous, rhombic-ovoid, trigonous, ca. 2 mm long, with three angles shallowly constricted at middle, sides slightly concave below, base with a short and curved stipe, apex abruptly contracted into a short, coiled beak; style base thickened; stigmas three.

#### Etymology.

The specific epithet “*liboensis*” refers to the type locality of this new species in Libo County of Guizhou Province.

#### Phenology.

Flowering and fruiting occur in late March.

#### Conservation status.

The new species is currently known only from the type locality. As surveys and collections at the type locality and in surrounding areas have been limited, its full geographic range, population size and trends, and potential threats remain insufficiently documented. Consequently, the available information is insufficient to determine whether the species meets any of the IUCN criteria for a threatened category. The species is therefore provisionally assessed as Data Deficient (DD) following the IUCN Red List Categories and Criteria (IUCN 2024).

#### Notes.

With its rhombic-ovoid nutlets and brown-green utricles (5–5.5 mm long), the new species is similar to *Carex austrosinensis* Tang & F.T.Wang ex S.Yun Liang. It differs from the latter in having rather densely flowered lateral spikes, peduncles enclosed in bract sheaths at the base, and nutlets shallowly constricted at the middle angles. In contrast, *Carex austrosinensis* possesses sparsely flowered lateral spikes, peduncles long-exserted from bract sheaths, and nutlets that are not constricted at the middle.

### 4. Carex canina

Taxon classificationPlantaePoalesCyperaceae

Dunn var. pubiutriculata Hong Wang var. nov.

7698FF92-5ED0-5CD7-A865-BA8884BF8BD3

urn:lsid:ipni.org:names:77484429-1

Figs 1F, 1G, 2M

#### Diagnosis.

The new variety differs from the autonymic variety in having pubescent utricles and sparsely pubescent bract sheaths.

#### Type.

China • Zhejiang: Longyou Xian, Miaoxia Town, near Fengwei Waterfall; alt. 480 m, in roadside grasses under bamboo forest; 13 May 2026; *H. Wang 1457* (holotype: ZJFC; isotype: ZM).

#### Etymology.

The variety epithet “*pubiutriculata*” refers to the utricles of this new variety being pubescent.

#### Phenology.

Flowering and fruiting occur in early to mid-May.

#### Additional specimens examined.

China • Zhejiang: Longyou Xian, Miaoxia Town, near Fengwei Waterfall, in roadside grasses under bamboo forest, 13 May 2026, *H. Wang 1454* (IBK, ZJFC, ZM); • the same locality, Qizixia, alt. 420 m, under bamboo forest, 9 May 2026, *H. Wang 1451* (ZJFC, ZM).

#### Conservation status.

Carex canina var. pubiutriculata is currently known from a few collections in Longyou County, Zhejiang Province, China. Comprehensive field surveys have not yet been conducted to determine its geographic range, population size and trends, or potential threats. The available information is therefore insufficient to determine whether the variety meets any of the IUCN criteria for a threatened category. The variety is provisionally assessed as Data Deficient (DD) following the IUCN Red List Categories and Criteria (IUCN 2024).

## Supplementary Material

XML Treatment for Carex zhangjiajieensis

XML Treatment for Carex glaucispicata

XML Treatment for Carex liboensis

XML Treatment for Carex canina

## Additional information

### Artificial Intelligence (AI) use

The authors accept full responsibility for the content of the manuscript, including the disclosure of any use of AI.

No AI tools were used in the preparation of this manuscript.

### Author ORCIDs

Y.-F. Lu https://orcid.org/0000-0001-6095-3821

D.-C. Meng https://orcid.org/0000-0002-1049-1232

X.-J. Zhao https://orcid.org/0000-0002-6251-5654

X.-F. Jin https://orcid.org/0000-0002-8195-6738

### Data availability

All of the data that support the findings of this study are available in the main text.
